# Alternative Risk Assessment for Dangerous Chemicals in South Korea Regulation: Comparing Three Modeling Programs

**DOI:** 10.3390/ijerph15081600

**Published:** 2018-07-28

**Authors:** Hyo Eun Lee, Jong-Ryeul Sohn, Sang-Hoon Byeon, Seok J. Yoon, Kyong Whan Moon

**Affiliations:** Department of Health Science, Korea University, Anam-ro 145, Seongbuk-gu, Seoul 02841, Korea; chokbab@naver.com (H.E.L.); sohn1956@korea.ac.kr (J.-R.S.); shbyeon@korea.ac.kr (S.-H.B.); ehslab@naver.com (S.-J.Y.)

**Keywords:** chemicals requiring preparation for accidents, risk assessment, ALOHA, KORA, PHAST, the Chemicals Control Act

## Abstract

Unlike other countries, the Korean chemical industry does not clearly distinguish between industrial sites and residential areas. The 2012 Gumi Hydrogen Fluoride Accident revealed that chemical accidents could cause damage to nearby residents. Accordingly, the Chemicals Control Act was enacted in 2015, which requested industrial sites using chemicals to perform a risk assessment for all chemical facilities and to distribute the results to the local residents and governments. Industrial businesses had the responsibility of warning the local residents. In this study, two programs (Areal Location of Hazardous Atmospheres (ALOHA), Process Hazard Analysis Software Tool (PHAST)) were compared with Korea Off-site Risk Assessment Supporting Tool (KORA), which is the current representative risk assessment program used in Korea Chemicals Control Act. The five chemical substances (nitric acid, hydrogen chloride, ammonia, sulfuric acid, and formaldehyde) most commonly involved in chemical accidents were selected. The range of influence of ERPG-2 (Emergency Response Planning Guideline) on chemical accidents was modeled and the results compared. ALOHA was found to be the most suitable program for the determination of toxicity for nitrate acid and ammonia, KORA for hydrogen chloride and sulfuric acid, and PHAST for formaldehyde. To maximize the safety of many local residents and to prepare for chemical accidents, risk assessments should be conducted using a variety of risk assessment programs, and the worst-case damage radius should be determined.

## 1. Introduction

Since the 1960s there has been social conflict and political confusion in Korea due to rapid industrialization and urbanization. In particular, indiscreet urban development has resulted in an unclear distinction between industrial sites and residential areas, causing continuous social conflicts between local residents and businesses [1]. The Gumi Hydrogen Fluoride Accident in 2012 is an example of this. Within 36 min of the chemical accident evacuations commenced for residents within a 50 m area, and after 4 h and 46 min evacuations were expanded to include all residents within a radius of 1.3 km. Five workers were killed at the scene of the accident, 18 people were killed in the neighborhood, including residents and workers, and a total of 12,243 were hospitalized. In addition, almost all vegetation within 1 km of the accident was affected, causing significant damage to commercial crops and livestock (compensation amounting to South Korean won (KRW) 38 billion) [2]. 

This incident demonstrated that chemical accidents not only affect the industrial workers, but also the surrounding communities and natural environment. Because of this accident, the Chemicals Control Act was born in Korea in 2015 and all workplaces that used chemicals were subject to legal restrictions. In the Chemicals Control Act, chemicals that were classified as toxic and dangerous substances were defined as ‘hazardous chemicals’, and detailed guidelines were presented [3]. However, there is always the risk of this type of accident reoccurring, as many businesses are already in close proximity to residential areas.

In the US (United States) and EU (European Union) the country-based industrial facilities and citizens’ residential complexes are clearly separated, and regulated through land use plans and laws such as the Seveso directive [4]. These countries aim to prevent chemical accidents through safety management and by ensuring sufficient distance from residential areas during the initial risk assessment when planning industrial building sites [5]. 

The Chemicals Control Act outlines how to assess the risk of any chemical facility by determining the risk of all chemicals in the workplace [6], and the scope of the worst damage is disclosed to local residents and workers of other companies [7]. The worst damage scenario is conservative yet is considered excessive legal regulation in Korea. However, the safety of the people is the most important value. The purpose of these Chemical Control Act may also apply to other countries. Residents who are within the scope of the worst damage scenario are informed about evacuation sites, first aid measures, and best evacuation practices (e.g., relocate to the center of a building, or evacuate to a higher ground) in the event of an accident [8].

In Korea, most of the modeling programs for evaluating the extent of damage use the KORA (Korea Off-site Risk Assessment Supporting Tool), which is provided by government agencies [9]. However, there are many kinds of modeling programs, and even when modeling with the same conditions, the damage radius results are different with different models [10]. 

As the Chemicals Control Act suggests only providing chemical accident information to residents in the worst potential damage area, if the damage radius distance differs from one modeling program to another, it may result in some people not receiving necessary information, which leaves them defenseless for when a chemical accident actually occurs [11].

In this study, we compare the results of three modeling programs by adding Areal Location of Hazardous Atmospheres (ALOHA) and Process Hazard Analysis Software Tool (PHAST) in addition to KORA, a modeling program used in Korea. In this way, we use various modeling programs to select the alternative risk assessment. 

## 2. Materials and Methods

### 2.1. Selected Chemical Substances

The Ministry of Environment of the Republic of Korea has provided various statistics on chemical accidents by the Chemical Safety Agency [12]. According to the number of chemical accidents that occurred during the period of 2015 to 2017, when the Chemicals Control Act was enacted, the top five substances were all classified as “Chemicals requiring preparation for accidents” (Table 1).

“Chemicals requiring preparation for accidents” are substances that have a high risk for causing accidents, or are likely to be harmful if accidents occur. The Chemicals Control Act defines them as the following:

- Highly hazardous substances with high flammability, potential for explosion, high reactivity, and possibility of leakage.
- Highly acute toxic substances when ingested, inhaled, or exposed to skin.
- Substances with a high risk of accident due to large amount of usage in Korea.
- Substances that are considered to require special management [13].

As of April 2018, there are 97 kinds of “chemicals requiring preparation for accidents”, and they are used throughout various industry processes. Chemicals requiring preparation for accidents are considered to have the greatest risk associated with them, as the probability of accident occurrence is high.

### 2.2. Selection of Chemical Facilities

Looking at statistics from 2014 to 2017, a total of 384 chemical accidents were reported. Of these, 96 cases occurred in storage tanks and 106 cases involved piping processes affecting storage tanks [12], resulting in a total of 52.6% of the cases related to storage tanks. Of the total number of accidents, 281 cases (73.1%) were caused by toxic leaks, and in 63 cases (16.4%) a fire explosion occurred. The average capacity of the storage tank in which accidents occurred was 10 m^3^. 

In this study, the materials and specifications of the storage tanks that were most likely to cause accidents were determined, and are presented in Table 2. The material used for each chemical was the average material used in the actual chemical factory [14]. The capacity of the tank was set to 10 m^3^, but assumed to be at approximately 80% capacity (8 m^3^), as tanks are rarely used in processing when entirely full. The endpoint concentration to be used in the damage radius estimation for each chemical substance was calculated by the Emergency Response Planning Guideline (ERPG-2) value [15]. ERPG-2 is the maximum airborne concentration below which nearly all individuals could be exposed for up to 1 h without experiencing or developing irreversible or other serious health effects that could impair an individual’s ability to take protective action [16].

The tank floor distance from the ground was 20 cm considering the height of the tank skirt when the storage tank was installed on the foundation. FRP (fiber reinforced plastic), which is a storage tank for hydrogen chloride, was estimated to be 10 cm since no separate skirt was installed (Table 2).

### 2.3. Modeling Program Selection

Five selected chemicals were modeled for toxic leaks based on a Gaussian model. When a chemical accident occurs, as in the case of the 2012 Gumi Hydrogen Fluoride Accident, fire explosions are usually isolated to the factory. However, a toxic leak can become widely spread depending on weather conditions, and can cause extensive damage to residential areas and residents. In order to investigate the leakage extent for each substance, we followed the “Technical Guidelines on the Selection of Accident Scenarios” provided by the Chemical Safety Agency [17].

The Areal Location of Hazardous Atmospheres (ALOHA) modeling program, provided by the US Environmental Protection Agency, was used. Further, in addition to the Korea Off-site Risk Assessment Supporting Tool (KORA), provided by Korea Chemical Safety Agency, the Process Hazard Analysis Tool (PHAST), which is famous for its commercial risk assessment program, was used (Table 3).

#### 2.3.1. ALOHA (Areal Location of Hazardous Atmosphere)

ALOHA was originally developed as a self-assessment tool by the National Oceanic and Atmospheric Administration’s (NOAA) Emergency Response Team, but it has been gradually improved and is now used in partnership with the US Environmental Protection Agency (EPA). It is a diffusion model, and the damage radius can be estimated in various scenarios. For example, by calculating how quickly chemicals escape from tanks, sumps, and gas pipelines, ALOHA predicts how the radius will change with the release rates. 

It is possible to derive the damage radius from many scenarios such as toxic leaks, jet fires, BLEVE (Boiling Liquid Expanding Vapor Explosions), and pool fires. The biggest advantage of ALOHA is its simplicity and ease of use [18].

#### 2.3.2. KORA (Korea Off-Site Risk Assessment Supporting Tool)

KORA is a modeling program distributed by the Korea Chemical Safety Agency. Based on a Gaussian model, it calculates the harmful chemical damage radius by toxic leaks and fire explosions. However, aqueous modeling such as that for sodium hydroxide is weak in KORA compared to ALOHA, because it is not estimated by the air diffusion model due to physicochemical characteristics. Recently, KORA’s function of deriving the damage distance by calculating the vapor pressure of the aqueous solution was added [9].

#### 2.3.3. PHAST (Process Hazard Analysis Software Tool)

PHAST, developed by DNV (Det Norske Veritas) Software, is a program that quantifies risk. It is designed to assess the progression of potential accidents, from initial leakage to diffusion, and the effects of explosions and toxicity. It is the world’s most widely used modeling program for risk assessment [19]. 

### 2.4. Weather Information

The meteorological conditions can influence modeling conditions. In this study, meteorological conditions were based on the Ulsan industrial complex (one of the biggest industrial complexes in Korea) (Table 4). The Chemicals Control Act regulates that modeling should be conducted based on an average year’s weather condition. But this study tried to analyze the changes according to the weather conditions by modeling program, divided into four seasons (spring, summer, fall, winter). In Korea, December to February are winter, March to May are spring, June to August are winter, and September to November are fall. The weather conditions for each season are shown in Table 5.

As of 2014, the Ulsan industrial complex has 1632 companies. Industrial facilities in Ulsan are concentrated, so there is a high possibility of a series of accidents due to explosion or fire. In addition, residential areas are often located near industrial facilities, which may cause damage to residents.

The Ministry of Environment’s Chemical Emissions and Transfer Information System discloses the amount of hazardous chemical substances released into the environment during the manufacturing process, as well as those that are stored in the workplace [20]. In 2013, Ulsan’s annual emission per unit area was the largest among the municipalities nationwide. Emissions from Ulsan National industrial complex were the largest among national industrial complexes in 2015 [21]. 

The meteorological stability is shown Table 6. According to The Chemicals Control Act, the atmospheric stability is modeled by calculating D (Meteorological stability in Table 6) under modeling conditions [17], and by referring to the yearbook of the Meteorological Agency, the monthly meteorological information was derived [22].

### 2.5. Operating Condition

In risk assessment, the amount of material is an important factor. The specific gravity of each substance was calculated and converted to a storage capacity (kg) [23]. In this study, chemicals in a storage tank were considered to have normal temperature and normal pressure conditions. The diameter of the pipe was 50 mm, therefore the leak hole was 50 mm. (Table 7)

### 2.6. Number of People Being Issued Chemical Accident Information

According to the Chemicals Control Act, local residents should be informed of the chemical accident risk and provided with an emergency response information summary. The summary includes basic information such as the location of business, contact information, the kinds of hazardous chemicals handled, and the hazards and risk of accidents. The most important information is the response information in case of an accident. It includes the status of the personal protective equipment and prevention equipment at the industrial factory, how to alert the local community and the surrounding workplaces, and how to prevent damage from the harmful chemicals. Information on this content is provided to local residents through written correspondence, public hearings, briefing sessions, and at local government offices [8]. All companies provide information summaries only to residents within the damage radius derived through the modeling program.

## 3. Results

### 3.1. Damage Distance for "Chemicals Requiring Preparation for Accidents"

The damage distance for each of the “chemicals requiring preparation for accidents” from the three modeling programs are shown in Table 8. Sulfuric acid modeling was not possible in the ALOHA program. All of the modeling programs were very different. ALOHA showed a big difference in damage distance by season. In the case of nitric acid, it was 482 m in spring but 1500 m in summer. On the other hand, there was little difference between seasons for hydrogen chloride, formaldehyde and ammonia. However, there was no change in the conditions of KORA by season. PHAST showed a little difference in damage distance by season. In the case of ALOHA, the damage distance for all chemicals was the greatest in summer. In contrast to ALOHA, PHAST produced damage distances that were greatest in spring. The damage distance radius of all chemicals showed a large difference by modeling program. In particular, the damage distance material ranking is different for each modeling program. The top three chemical substances by damage distance are different for all modeling programs. KORA showed the greatest damage distance for hydrogen chloride, followed by ammonia and formaldehyde. ALOHA had the greatest damage distance of ammonia, second was nitric acid, and third was hydrogen chloride. PHAST, like KORA, showed the greatest damage distance of hydrogen chloride, second was formaldehyde, and third was nitric acid (Table 9). 

### 3.2. Selected Modeling Program

**Figure 1:**
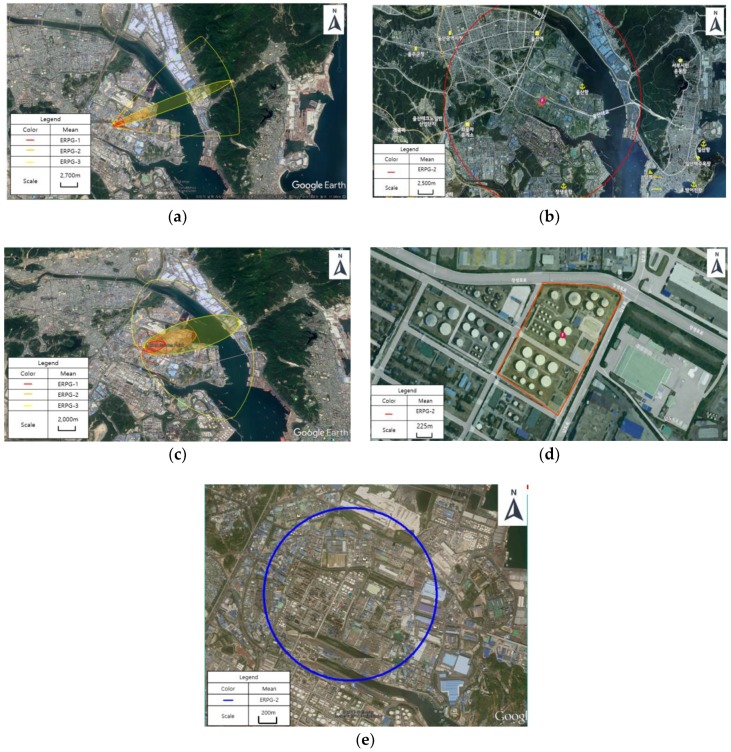
The largest damage distance for the modeling program: (**a**) Nitric acid damage distance (1500.0 m)—ALOHA; (**b**) Hydrogen chloride damage distance (4340.5 m)—KORA; (**c**) Ammonia damage distance (2100.0 m)—ALOHA; (**d**) Sulfuric acid damage distance (7.8 m)—KORA; and (**e**) Formaldehyde damage distance (888.7 m)—PHAST.

A chemical accident scenario should be derived from a modeling program that represents the worst damage distance for each substance. The results for “chemicals requiring preparation for accidents” with the worst damage distance for each modeling program were plotted (Figure 1). Various results were obtained from each modeling program. This result was predictable based on several previous studies [10]. ALOHA program can represent three endpoint concentrations simultaneously. In Figure 1a,c, each color represents ERPG-1,2,3. (Red: ERPG-3, Orange: ERPG-2, Yellow: ERPG-1). The reference concentration in this study is the orange distance because it is the ERPG-2 value. The modeling point is there Ulsan industrial complex and the coordinates are 35,305,204 in the north and 129,212,291 in the east. Weather condition (summer) is 25.3 °C, humidity is 66%. Wind direction is WSW and wind speed is 2.1 m/s for case (a) and (c). KORA program has an average annual weather condition of 24.2 °C, humidity is 59%. Wind direction is WNW and wind speed is 2.3 for case (b) and (d) because there is no difference in weather conditions. Weather condition (spring) is 13.5 °C, humidity is 54%. Wind direction is WNW and wind speed is 2.4 m/s for case (e).

### 3.3. Modeling Program for the Number of People Provided with Chemical Accident Information

KORA is the recommended modeling program in the Korea Chemicals Control Act. Therefore, most Korean workplaces use KORA to calculate the damage distance in order to establish countermeasures against chemical accidents. As KORA is the standard modeling program, the results of the other modeling programs were compared against it. Assuming KORA’s damage radius to be 1, the results from the other modeling programs were used to determine how different they were from KORA through ratios. The results (Table 10) show that there are some differences between the modeling programs.

The results indicate that KORA could be used for chemical accidents if the damage distance is higher than other modeling programs. However, if other modeling programs show larger values, it suggests that an alternative risk assessment is needed.

**Figure 2:**
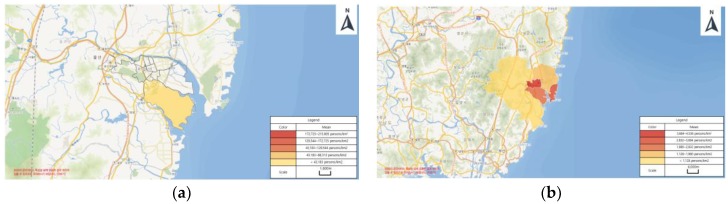
The population density: (**a**) Ulsan industrial complex (43,183 persons/km^2^); (**b**) Ulsan average density (1099 person/km^2^).

The population density of the Ulsan industrial complex area was used to estimate the number of people who would need to be provided with chemical accident information (Figure 2) [24]. In the Chemical Controls Act, residents outside the workplace boundaries are surveyed by visit or phone to calculate the exact number of people who will affected chemical accident. The number of people determined by the modeling programs varied greatly. The chemical requiring preparation for accidents with the greatest differences was hydrogen chloride. Table 11 shows the results of the KORA damage extent prediction, indicating 2,555,183 people in the surrounding area would require a chemical accident summary.

## 4. Discussion

Sulfuric acid was not modeled in the ALOHA program because while it is dangerous to inhale, it is not likely to spread quickly enough in dangerous concentrations to people in large areas under normal conditions. This is due to the low vapor pressure and low volatility of sulfuric acid when dispersed through the air into the atmosphere. It has a very low level of vapor pressure of only 1 millimeter (mm H_2_O) at 146 °C, compared to water under the same conditions having a vapor pressure of 44,000 millimeter (mm H_2_O) [25,26]. The reason why the ALOHA program has the largest damage distance in the summer conditions is that the temperature is high and the atmospheric diffusion of the chemical is expected to be faster.

The ALOHA program has shown great toxic effects in nitric acid and ammonia. In 1996 the ALOHA program versioning was modified to account for the surface tension among the physical properties related to ammonia. In 2009, ALOHA was updated for the reactivity and carcinogenicity of nitric acid. There were also updates made using results from field tests on the marine transport tank conducted by the US Navy in relation to hydrochloric acid and ammonia [27]. Due to the history of modeling program-specific updates, different results for different “chemicals requiring preparation for accidents” were expected. This update history would have caused the nitric acid and ammonia damage distance to be large in ALOHA. The technical limitations of the ALOHA program are influenced by area and building-related impacts. If the surrounding area is a highly dense environment, such as with many buildings, actual gas diffusion is prevented. However, there is no method to incorporate this factor into the ALOHA program. Furthermore, ALOHA should not be used when the wind speed is less than 1 m/s. In addition, it is impossible to model the reaction that occurs when a chemical leaks into the air and reacts with the atmosphere [28]. Comparatively, PHAST is a more realistic physical model, in that topography factors can be selected and the setting of the initial leak rate can be applied [29]. However, PHAST is also similar to ALOHA with building-related impacts. PHAST does not consider high building density and the environment when modeling. The reason for the largest damage distance in spring from PHAST seems to be a combined factor for wind speed and temperature. 

KORA is a recently developed program compared to the other two modeling programs (ALOHA: 1980s, PHAST: 1990s, KORA: 2015). Hydrogen chloride has a large damage distance, which is predicted to be similar for other chemicals (e.g., hydrogen fluoride or hydrogen peroxide) due to the after effect of the 2012 Gumi Hydrogen Fluoride Accident. In fact, hydrogen chloride is a substance with a high vapor pressure (40.5 times the atmospheric pressure), which means that the surface of the leaking hydrogen chloride liquid evaporates and vaporizes well. If the leakage area is wider than the modeled condition, the damage distance may be much larger. According to the development background and update records of the three modeling programs, damage radius distance is considered to be different for each modeling program. 

KORA is the recommended modeling program in the Korea Chemicals Control Act. KORA had no change in the damage distance based on the weather (spring, summer, fall, winter) condition. This could be a weakness in the modeling program. However, because the Korean Chemicals Control Act progresses the modeling through the annual average weather information, an important modeling factor in KORA is the chemical’s physicochemical properties and storage conditions rather than weather information.

However, the weather environment is an important factor in a damage prediction model. Modeling with annual averages in the Chemicals Control Act should be modified. Especially in Korea, it is reasonable to model by season because Korea’s four seasons are distinct. By comparing KORA with other modeling programs, it is possible to predict which of the five types of “chemicals requiring preparation for accidents” may be mismanaged in the event of a chemical accident. In this study, there are potential problems in the case of nitric acid, ammonia, and formaldehyde. In the case of nitric acid, the damage distance of ALOHA was 14 times higher than that of KORA. Also, ammonia’s damage distance was more than two times higher in the ALOHA scenario than that in KORA. Formaldehyde had a PHAST value nearly three times higher than that of KORA. This suggests that using KORA alone as a modeling program to prevent and respond to chemical accidents in Korea is problematic. 

In the case of hydrogen chloride, the extent of the KORA damage predictions results in issuing chemical accident summaries to 2,555,183 people in the surrounding area. This means that not only information summaries are transmitted, but also that various social network infrastructures are required from the local governments (e.g., city hall or county offices), fire departments, police stations, and hospitals to support injured people. Conversely, when modeling with ALOHA or PHAST, support for 10,032 and 159,920 people is relatively low. The difference in the number of people is likely to lead to a larger distribution gap for industrial factories in areas with higher population density. Also, by showing different results for different modeling programs, it demonstrates that the modeling program cannot know exactly how many people will actually be affected by a chemical accident. However, in order to take the highest precautions against chemical accidents, many modeling program predictions are required and must be properly managed.

The modeling results may be different from the diffusion results in real-life. This is a limitation of modeling. If possible, it is also necessary to compare the modeling values through field experiments [30].

## 5. Conclusions

In this study, risk assessment was performed using three modeling programs for each of the top five “chemicals requiring preparation for accidents”. It was determined that the modeling program best suited for nitric acid and ammonia accidents was ALOHA, while the KORA program was superior for hydrogen chloride and sulfuric acid accidents, and PHAST for formaldehyde accidents.

The KORA program was seen as an exaggerated result for hydrogen chloride. Materials with high vapor pressure such as hydrogen peroxide and hydrogen fluoride can make exaggerated results in KORA because the Gumi Hydrogen Fluoride accident occurred in Korea. Hydrogen fluoride has a high vapor pressure, therefore, other chemicals having similar physicochemical properties can also cause an accident similar to the Gumi Hydrogen Fluoride accident. Currently, KORA is most commonly used in the Chemicals Control Act assessments, wherein the most dangerous substances are reported as nitric acid, ammonia, formaldehyde, and hydrogen chloride; and these are regarded as exaggerated evaluations. However, most workplaces in the Republic of Korea are located close to residential areas. The Chemical Control Act assumes civilians and workers within the damage radius determined by KORA to be potential victims of chemical accidents. These people are provided with a summary of chemical accident information. 

In order to prevent chemical accidents, it is necessary to give as many people as possible warning about the possibility of an accident, and provide evacuation tips in the event of an accident. To do so, a chemical accident scenario derived from a modeling program that represents the worst damage distance should be evaluated and compared against other modeling programs. 

## Figures and Tables

**Table 1:** The number of accidents by “chemicals requiring preparation for accidents” during 1 January 2015–31 December 2017.

Rank	Chemical (CAS No. ^1^)	Number of Accidents (Ratio)
1	Nitric Acid (7697-37-2)	29 (16.5%)
2	Hydrogen Chloride (7647-01-0)	19 (10.8%)
3	Ammonia (7664-41-7)	18 (10.2%)
4	Sulfuric Acid (7664-93-9)	16 (9.1%)
5	Formaldehyde (50-00-0)	14 (8.0%)

^1^ CAS No: Chemical Abstracts Service Registration Number.

**Table 2:** Storage tank material and specifications for “chemicals requiring preparation for accidents”.

Rank	Chemical (CAS No.)	Material	Capacity (m^3^)		Dike	ERPG-2 ^1^ (ppm)	Distance from the Ground (m)
			Design (mm)	Operation			
1	Nitric Acid (7697-37-2)	STS 304 ^2^	10 (D ^3^: 2300; H ^4^: 2700)	8	Horizontal 5300 mm; Vertical 5300 mm; Area 23.94 m^2^; Volume 14.05 m^3^	10	0.2
2	Hydrogen chloride (7647-01-0)	FRP ^5^	10 (D ^3^: 2300; H ^4^: 2700)	8		20	0.1
3	Ammonia (7664-41-7)	STS 304 ^2^	10 (D ^3^: 2300; H ^4^: 2700)	8		150	0.2
4	Sulfuric Acid (7664-93-9)	STS 304 ^2^	10 (D ^3^: 2300; H ^4^: 2700)	8		2.49	0.2
5	Formaldehyde (50-00-0)	STS 304 ^2^	10 (D ^3^: 2300; H ^4^: 2700)	8		10	0.2

^1^ ERPG-2: Emergency Response Planning Guideline, ^2^ STS 304: stainless steel material, ^3^ D: diameter, ^4^ H: height, ^5^ FRP: fiber reinforced plastic.

**Table 3:** Characteristics of three modeling programs.

Title	ALOHA	KORA	PHAST
Driving model	Gaussian atmospheric diffusion	Gaussian atmospheric diffusion	Discharge model, Multi component extension model, dispersion model, etc.
Chemicals data base	1000 chemicals	1000 chemicals	1000 chemicals
Mixed substance	modeling impossible	modeling impossible	modeling possible
Major Usage	Derives a simple result for a variety of uses	For compliance with Korea regulations	Widely used for commercial use
Mapping	Google Earth (Worldwide)	V-world (Korea map)	Google Earth (Worldwide)
Advantages	Free (US EPA), provides quick results	Free (Korea Ministry of Environment), provides quick results	Scenario based case-expandable, considers the surface, can be displayed in three-dimensions (3D)
Disadvantages	Impossible to calculate three-dimensional concentration distribution, impossible to realize atmospheric chemical reaction, prediction to 10 m	Decreased accuracy of aqueous solutions, little fluctuation in seasonal conditions, impossible to calculate three-dimensional concentration distribution	The price is very expensive

ALOHA = Areal Location of Hazardous Atmospheres; KORA = Korea Off-site Risk Assessment Supporting Tool; PHAST = Process Hazard Analysis Software Tool; US EPA = United States’ Environmental Protection Agency.

**Table 4:** Operating meteorological conditions for month (Ulsan industrial complex).

Month	Average Temperature (°C)	Average Humidity (%)	Main Wind Direction	Average Wind Speed (m/s)
1	−3.2	53	N	2.5
2	2.0	52	NW	2.8
3	7.0	51	NW	2.4
4	14.1	55	WNW	2.4
5	19.5	56	WNW	2.4
6	23.3	63	WSW	2.1
7	24.5	73	WSW	2.1
8	28.0	64	ENE	2.1
9	23.1	65	S	2.0
10	16.1	62	NNE	2.1
11	6.8	57	S	2.2
12	1.2	59	W	2.2
Average	24.2	59	WNW	2.3

**Table 5:** Operating meteorological conditions for season (Ulsan industrial complex).

Season	Average Temperature (°C)	Average Humidity (%)	Main Wind Direction	Average Wind Speed (m/s)
Spring	13.5	54	WNW	2.4
Summer	25.3	66	WSW	2.1
Fall	15.3	61	S	2.2
Winter	0	54	N	2.5

**Table 6:** Meteorological stability for Pasquill’s classes.

Wind Speed (m/s)	Day Time			Night Time	
	Size of Radiation Intensity				
	Strong	Moderate	Slight	Cloudy	Sunny
<2	A	A–B	B	F	F
2~3	A–B	B	C	E	F
3~5	B	B–C	C	D	E
5~6	C	C–D	D	D	D
6>	C	D	D	D	D

A: Very unstable, B: Instability, C: Slight instability, D: Neutral, E: Slightly stable, F: Very stable.

**Table 7:** Operating and maintaining conditions of storage tank.

Rank	Chemical (CAS No.)	Capacity (m^3^)		Operating Condition		Specific Gravity	Storage (kg)	Phase	Hole (mm)
		Design	Operation	Temperature	Pressure				
1	Nitric Acid (7697-37-2)	10	8	AMB ^1^	ATM ^2^	1.52	12,160	Liquid	50
2	Hydrogen chloride (7647-01-0)	10	8	AMB ^1^	ATM ^2^	1.6	10,080	Liquid	50
3	Ammonia (7664-41-7)	10	8	AMB ^1^	ATM ^2^	0.8	4640	Liquid	50
4	Sulfuric Acid (7664-93-9)	10	8	AMB ^1^	ATM ^2^	1.83	14,640	Liquid	50
5	Formaldehyde (50-00-0)	10	8	AMB ^1^	ATM ^2^	0.82	6560	Liquid	50

^1^ AMB: ambient (outside temperature), ^2^ ATM: atmospheric pressure (normal pressure).

**Table 8:** Seasonal damage distances by three modeling programs.

No.	Chemical (CAS No.)	KORA Distance (m)	ALOHA Distance (m)				PHAST Distance (m)			
		Same as Season	Spring	Summer	Fall	Winter	Spring	Summer	Fall	Winter
1	Nitric Acid 7697-37-2)	104.2	482	1500	839	515	334.2	333.1	343.1	330.7
2	Hydrogen chloride (7647-01-0)	4340.5	215	572	537	205	1028.2	1001.5	956	959
3	Ammonia (7664-41-7)	882.7	1900	2100	1900	1900	285.2	282.3	267.8	263.9
4	Sulfuric Acid (7664-93-9)	7.8	-	-	-	-	2.59	2.6	2.59	2.58
5	Formaldehyde (50-00-0)	338.5	240	257	239	210	888.7	875.6	829	827.9

**Table 9:** A comparison of each modeling program distances in the worst case scenarios.

Rank	Chemical (CAS No.)	KORA Distance (m)	ALOHA Distance (m)	PHAST Distance (m)	KORA Rank	ALOHA Rank	PHAST Rank
1	Nitric Acid (7697-37-2)	104.2	1500.0	343.1	4	2	3
2	Hydrogen chloride (7647-01-0)	4340.5	572.0	1028.2	1	3	1
3	Ammonia (7664-41-7)	882.7	2100.0	285.2	2	1	4
4	Sulfuric Acid (7664-93-9)	7.8	-	2.59	5	5	5
5	Formaldehyde (50-00-0)	338.5	257.0	888.7	3	4	2

**Table 10:** Damage distance compared ratio based on KORA.

Rank	Chemical (CAS No.)	KORA Standard	ALHOA Ratio	PHAST Ratio
1	Nitric Acid (7697-37-2)	1	14.39	3.16
2	Hydrogen chloride (7647-01-0)	1	0.062	0.25
3	Ammonia (7664-41-7)	1	2.38	0.31
4	Sulfuric Acid (7664-93-9)	1	-	0.33
5	Formaldehyde (50-00-0)	1	0.72	2.62

**Table 11:** Number of people expected to be prepared for an accident.

Rank	Chemical (CAS No.)	KORA (People)	ALHOA (People)	PHAST (People)
1	Nitric Acid (7697-37-2)	1467	305,088	14,677
2	Hydrogen chloride (7647-01-0)	2,555,183	10,032	159,920
3	Ammonia (7664-41-7)	105,722	597,972	9666
4	Sulfuric Acid (7664-93-9)	9	-	1 ^1^
5	Formaldehyde (50-00-0)	15,583	8007	107,163

^1^ Calculated as 1 for less than 1 person.

## References

[1] (2013). A Study on Off-Site Consequence Analysis by Risk Management of Chemical Plant. http://www.riss.kr/link?id=A103038791.

[2] Gu S., Choi I. (2013). Study on the Distribution of Fluorides in Plants and the Estimation of Ambient Concentration of Hydrogen Fluoride Around the Area of the Accidental Release of Hydrogen Fluoride in Gumi. J. Environ. Health Sci..

[3] National Law Information Center Chemicals Control Act. http://www.law.go.kr/.

[4] Byoung-Hyo M. (2015). Seveso-Richtlinie III und Risiko Management System.

[5] Christou M., Biermann S.T. (2006). Land Use Planning Guidelines in the Context of Article 12 of the Seveso II Directive 96/82/EC as Amended by Directive 105/2003/EC.

[6] Lee G.-J., Ho J.S. (2017). A Study on Off-Site Risk Assessment of 10 Representative Chemicals in Chemical Industry.

[7] Chemical Information System. http://icis.me.go.kr/main.do.

[8] National Law Information Center Chemicals Control Act Article 42 (Notice of Risk Management Plans to Local Communities). http://www.law.go.kr/.

[9] Kim J.K., Ryu J.S. (2018). Suggestions for Increasing Utilization of KORA for Supporting the Off-site Risk Assessment System. J. Environ. Health Sci..

[10] Hannaa S., Dharmavaram S. (2008). Comparison of Six Widely-Used Dense Gas Dispersion Models for Three Recent Chlorine Railcar Accidents.

[11] National Law Information Center Chemicals Control Act Article 1 (Purpose). http://www.law.go.kr/.

[12] CSC Chemistry Safety Clearing-House. https://csc.me.go.kr/.

[13] National Law Information Center Chemicals Control Act Article 39 (Designation of Chemicals Requiring Preparation for Accidents). http://www.law.go.kr/.

[14] Korea Standard Association (2016). Construction of Welded Steel Tanks for Oil Storage KS6225.

[15] Jung Y.K., Kim B. (2017). A Study on the Simplified Estimating Method of Off-site Consequence Analysis by Concentration of Hydrochloric Acid. J. Korean Soc. Saf..

[16] Emergency Response Planning Guidelines. https://response.restoration.noaa.gov/oil-and-chemical-spills/chemical-spills/resources/emergency-response-planning-guidelines-erpgs.html.

[17] Chemical Safety Agency (2014). Technical Guidelines for Selection of Accident Scenario.

[18] NOAA’s National Ocean Service Office of Response and Restoration ALOHA Fact Sheet. http://response.restoration.noaa.gov.

[19] DNV GL AS Software PHAST. www.dnvgl.com/software.

[20] Pollutant Release and Transfer Register. http://icis.me.go.kr/prtr/main.do.

[21] Kim S.-J., Kwon H.-O. (2016). Selection of Priority Chemicals and Areas for the Response to Chemical Accidents in National Industrial Complexes. J. Korean Soc. Environ. Anal..

[22] Korea Meteorological Administration (2016). Annual Climatological Report.

[23] KOSHA Chemical Material Information. http://msds.kosha.or.kr/.

[24] Statistic Korea. http://www.index.go.kr/.

[25] Frequently Asked Questions (FAQs) about ALOHA. https://response.restoration.noaa.gov/oil-and-chemical-spills/chemical-spills/response-tools/aloha-faqs.html.

[26] Green D.W., Perry R.H. (2007). Perry’s Chemical Engineer’s Handbook.

[27] ALOHA Development History. https://response.restoration.noaa.gov/oil-and-chemical-spills/chemical-spills/response-tools/aloha-development-history.html.

[28] Jones R., Lehr W. (2013). ALOHA^®^ (Areal Locations of Hazardous Atmospheres) Technical Documentation.

[29] Kim H.S., Jeon B.H. (2017). Analysis of Impact Zone of Quantitative Risk Assessment based on Accident Scenarios by Meteorological Factors. J. Korean Soc. Environ. Eng..

[30] Hanna S.R., Strimaits D.G., Chang J.C. (1991). Evaluation of fourteen hazardous gas models with ammonia and hydrogen fluoride field data. J. Hazard. Mater..

